# Social tipping points in animal societies

**DOI:** 10.1098/rspb.2018.1282

**Published:** 2018-09-19

**Authors:** Jonathan N. Pruitt, Andrew Berdahl, Christina Riehl, Noa Pinter-Wollman, Holly V. Moeller, Elizabeth G. Pringle, Lucy M. Aplin, Elva J. H. Robinson, Jacopo Grilli, Pamela Yeh, Van M. Savage, Michael H. Price, Joshua Garland, Ian C. Gilby, Margaret C. Crofoot, Grant N. Doering, Elizabeth A. Hobson

**Affiliations:** 1 Department of Ecology, Evolution and Marine Biology, University of California – Santa Barbara, Santa Barbara, CA 93106, USA; 2 Department of Psychology, Neuroscience and Behaviour, McMaster University, Hamilton, Ontario L8S 4K1, Canada; 3 School of Aquatic and Fisheries Sciences, University of Washington, Seattle, WA 98195, USA; 4 Santa Fe Institute, Santa Fe, NM 87501, USA; 5 Department of Ecology and Evolutionary Biology, Princeton University, Princeton, NJ 08544, USA; 6 Department of Ecology and Evolutionary Biology, University of California – Los Angeles, Los Angeles, CA 90095, USA; 7 Department of Biology, University of Nevada – Reno, Reno, NV 89557, USA; 8 Edward Grey Institute of Field Ornithology, Department of Zoology, University of Oxford, Oxford OX1 3PS, UK; 9 Cognitive and Cultural Ecology Research Group, Max Planck Institute of Ornithology, Radolfzell, 78315, Germany; 10 Department of Biology, University of York, Heslington, York YO10 5DD, UK; 11 School of Human Evolution and Social Change, and Institute of Human Origins, Arizona State University, Tempe, AZ 85287, USA; 12 Department of Anthropology, University of California Davis, Davis, CA 95616, USA

**Keywords:** complex system, collapse, cooperation, critical point, hysteresis, social network

## Abstract

Animal social groups are complex systems that are likely to exhibit tipping points—which are defined as drastic shifts in the dynamics of systems that arise from small changes in environmental conditions—yet this concept has not been carefully applied to these systems. Here, we summarize the concepts behind tipping points and describe instances in which they are likely to occur in animal societies. We also offer ways in which the study of social tipping points can open up new lines of inquiry in behavioural ecology and generate novel questions, methods, and approaches in animal behaviour and other fields, including community and ecosystem ecology. While some behaviours of living systems are hard to predict, we argue that probing tipping points across animal societies and across tiers of biological organization—populations, communities, ecosystems—may help to reveal principles that transcend traditional disciplinary boundaries.

## 1. Introduction

Many animals are social, and behaviours that occur within social groups can affect individuals, their immediate neighbours, and the overall performance of the society. In some cases, even small changes in external environmental conditions can cause large and abrupt changes to individuals' behaviours, interactions among group members, and therefore how the group functions as a whole. Examples of changing environmental conditions include food deprivation, heat/cold stress, predation risk, or various anthropogenic stressors. Uncovering how and why small perturbations can cause marked and abrupt shifts in group dynamics is important for understanding group functioning, cohesion, and responsiveness to the environment. Here, we introduce the idea of tipping points, which have been used to better understand the dynamics of complex systems in many fields.

The term *tipping point* was first used in the academic literature by Morton Grodzins to describe racial segregation in US cities [1]. Ecologists and climate scientists have since used tipping points to better understand shifts in lake eutrophication [2], forest-grass transitions [3], and coral reef states [4]. Although the idea of tipping points has been used as a popular analogy for sudden changes in social systems, the conceptual framework underlying tipping points has not been widely applied to questions in behavioural ecology. In this article we explain what tipping points are, how they have been studied in other contexts, and how the tipping point framework could provide new insights and predictive power into the study of animal behaviour.

### (a) What are tipping points?

Tipping points are drastic shifts in the behaviour of systems as a result of small changes to the environment. In ecology, tipping points are often referred to as *ecological thresholds* [5–7]. For example, a small change in the temperature of a lake can lead to large shifts in the composition of the lake's community. Other commonly cited examples of ecological tipping points include sudden shifts in species dominance or population collapse [8,9].

Similarly, in a social context, *social tipping points* occur when small changes to the physical or social environment result in qualitative changes to group behaviour or dynamics [10]. In animal societies, tipping points could be used to explain social transitions such as the onset of collective movements, shifts in group behaviour from calm to agitated states, the emergence and disappearance of wars between neighbouring societies, the formation or disbandment of cooperation, or the diffusion of new innovations. For instance, African desert locusts rapidly shift between their little-observed solitary state to a swarming plague phenotype. The transition between these states is density-mediated and catalysed by positive feedback loops between population density, individual activity level, and serotonin-mediated gregariousness [11–13]. Thus, small changes in population density can cause large and abrupt changes in both individual state and group dynamics in these locusts.

## 2. Core concepts of social tipping points

**Figure 1.**
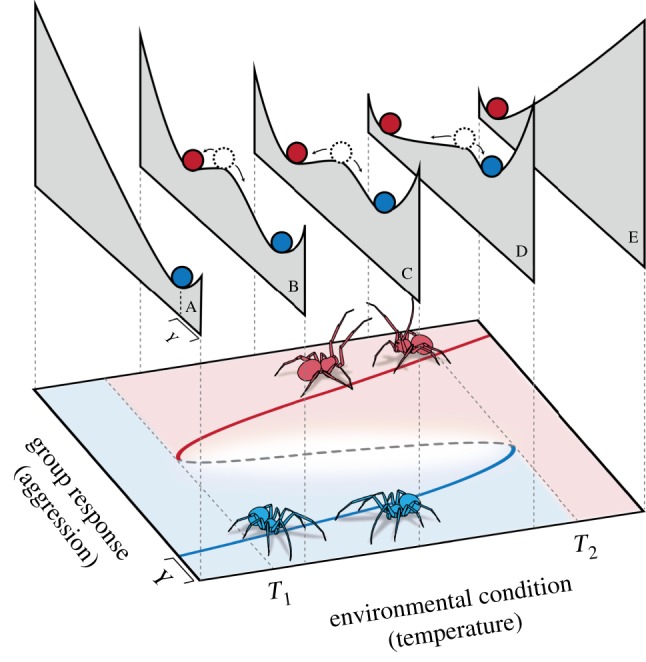
A hysteresis window between an environmental condition (e.g. temperature) and group behaviour (e.g. degree of infighting). This figure is modelled after a study on within-group conflict in response to heat stress in social spiders. Groups that have been in an agitated state (red) tend to remain agitated, whereas calm groups (blue) tend to remain calm. Therefore, there exists a set of intermediate environmental conditions (*T*_1_ < *T* < *T*_2_) where a group can be either calm or agitated depending on its historical dynamics. In the lower panel, solid lines represent stable equilibria states and the shaded regions show their basins of attraction. The dashed line is an unstable equilibrium, which demarks the boundary between the basins of attraction. The upper panels (A–E) provide an alternate abstraction of this system: for a given environmental condition, the group response tends to a low point on the ‘landscape’. The bottoms of the troughs in the upper panels are therefore stable equilibria and correspond to the locations of the solid red and blue lines in the lower panel (see ‘*Y*’ label for an example). Tipping points occur when a stable equilibrium (solid line/trough) collides with an unstable equilibrium (dashed line/peak) and is eliminated—at this point the system transitions suddenly to the alternate remaining equilibrium. In this system the tipping points are at *T*_1_ (when the system is in the agitated state and temperature is decreasing) and at *T*_2_ (when the system is in the calm state and temperature is increasing).

**Figure 2.**
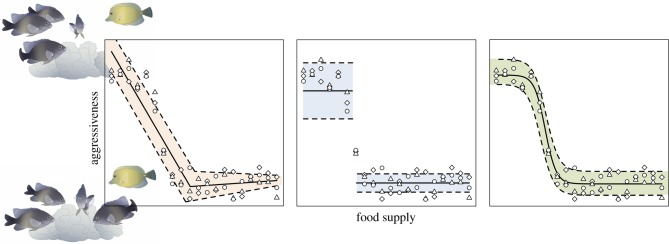
Social tipping points are characterized by an abrupt change in behaviour state caused by small changes in environmental parameters. Here, groups of territorial damselfish (brown fishes) may respond with vigilance and inspection (top image) towards intruders or not (bottom image) depending on whether food is limited. One sign of a possible tipping point is a change point in the data, where the data suddenly appear to be nonstationary. In the plot, this is depicted as a sudden change in the mean of aggressiveness (*y*-axis). If a model for aggressiveness is built for conditions where food supply is low, but then applied to cases where food supply is high, the model will have very large error. This reinforces the point that the old model is no longer valid for the new data if a tipping point has occurred. The three functions fitted to the identical data above have all been used to estimate the position of tipping points along environmental gradients, though the centre panel reinforces the point that entirely new models may be required to explain system properties before versus after a tipping point.

There are several concepts that are needed to apply the conceptual framework of tipping points to social systems. We describe these concepts here using an example. Social spider colonies exhibit a tipping point towards violent infighting in response to heat stress (figure 1) [14]. When colonies have been in cool temperatures (less than 27°C) they are generally calm and cooperative but transition into infighting at higher temperatures (greater than 31°C). However, when the temperature cools, colonies do not immediately return to their calm state upon reaching the critical 30–31°C, but require much cooler temperatures (less than 27–28°C) to return to their prior state. Thus, at an equivalent temperature, say 29°C, a colony can be characterized by high levels of infighting or calm cooperation, depending on its history. Notably, the shift between calm and agitated colony behaviour is mediated by temperature (the external *environmental parameter*), and this shift is conspicuously abrupt, which is diagnostic of social tipping points (figure 2).

### (a) Behavioural states and environmental parameters

Many animal social systems are capable of exhibiting multiple qualitatively distinct states. We refer to these as *behavioural states*, such as the calm (blue) and agitated (red) colony states in the spiders in figure 1. The behavioural state expressed is dictated by the system's dynamics as well as *environmental parameters* such as humidity or temperature (figure 1, *x*-axis) and *internal parameters* such as metabolic or cognitive factors. For social tipping points, we deem forces acting from outside the group to be *environmental parameters* and forces emerging from within the group as *internal parameters*.

Environmental parameters can be abiotic or biotic. Most studies on tipping points have examined abiotic drivers [3,6,14], whereas relatively few have examined biotic drivers, social or otherwise. Abiotic parameters include temperature, light, precipitation, oxygen levels, pH, aridity, anthropogenic noise, tides, and terrain [3,6,8]. Biotic parameters can be social (e.g. the number or collective phenotypes of nearby groups) or nonsocial (e.g. predation threat, food availability, or presence of parasites/disease). It is worth noting that many tipping points may be driven by changes in several environmental parameters, such as the combination of heat and UV exposure. Because of the potential combined effects, it is important to consider to what degree phenomena like priming, enhanced lethality of multiple stressors, or cross-tolerance affect group behaviour [15–18]. Multiple interacting environmental parameters could be grouped into functionally similar groups based on their properties or because of the shared effects that they have on social groups.

### (b) Attractors and basins of attraction

Up to this point we have presented behavioural states as categorical (such as calm and agitated), but behaviours can actually be more fluid. For example, a spider may be slightly irritated, but not fully agitated. As time progresses, the spider may become calm or agitated, depending on environmental parameters. In this example, the categorical states of calm and agitated are referred to as *attractors* and the set of fluid states that tend towards these categorical states are these attractors' *basins of attraction*. In figure 1, the solid red and blue lines depict the agitated and calm attractors for a range of environmental parameters (here, temperature). The lighter shaded areas in figure 1 are the basins of attraction for these two attractors. For intermediate environmental parameters, two attractors exist. At very low temperatures, there is only one attractor, the calm state, while at very high temperatures, only the agitated attractor exists. It is important to emphasize that attractors can appear and disappear, depending on environmental parameters.

In some cases, environmentally driven tipping points may be irreversible. For example, events such as the onset of sex change in sequential hermaphrodites [19], the onset of epidemic spawning in marine invertebrates [20], or the emergence of sexual alates in social insects [21] can be one-way transitions in behavioural state driven by minor perturbations to environmental parameters. In these cases, the former attractors have vanished as a consequence of the system undergoing a tipping point.

In addition to the presence and number of attractors, the landscape of attraction can vary. In figure 1, this is depicted with the landscape slices above the main figure which show how the geometry of the basins of attraction are modified as environmental parameters change. In each case, the blue or red balls indicate the attractors at the bottom of wells symbolizing the basins of attraction. The steepness of the walls of these basins of attraction determine the strength of the feedback mechanisms keeping the system in a given state—the steeper the walls of the wells, the quicker the system returns to the attractor state and the more resistant the state is to noise. When the wells are shallow, the system returns to the attractor more slowly and drifts more widely in response to noise [9,22,23].

### (c) Perturbations

There are two fundamentally different ways that a system can be perturbed. Either the behavioural state or the environmental parameters can be perturbed. To think about the effect of perturbations to the behavioural state, consider a single slice of the landscape of attractors in figure 1. When the behavioural state is perturbed, envision the system as one of the coloured balls that are subject to that particular landscape. If the ball is perturbed enough that it moves to another basin of attraction then the system undergoes a behavioural state change. However, this kind of perturbation is not technically classified as a tipping point because the transition was not caused by changes to the external environment. In contrast, when an environmental parameter changes, the landscape itself changes, which can alter the existence of attractors and the shapes of their basins of attraction. In figure 1, this is depicted by the series of slices showing the landscapes governing the basins of attraction. The society moves through a tipping point when a small change to environmental parameters results in a drastic enough modification to the attractor landscape that the society is now in an alternative basin of attraction. A critical difference between the two types of perturbations is that when a tipping point occurs, the underlying dynamics have changed and thus the previous regime's models and data are no longer effective in describing the new regime.

Attractor states are not necessarily advantageous or disadvantageous. For example, social groups might proceed from a relatively calm cooperative stable state to disbandment or collapse due to infighting or cheating [14,24]. However, a system might also switch between two states that perform equally well. The alternative states might even be part of a system's life history. Thus, attractor states are not necessarily evolutionary stable states (ESS) nor adaptive peaks in a fitness landscape, nor do they necessarily have negative consequences for social groups.

## 3. Tipping points: frequently asked questions

### (a) How can we recognize tipping points?

It can be difficult to recognize that a tipping point has occurred from observational data alone, especially if observations are noisy. However, there are some signatures of tipping points that one may recognize in their system of interest. One signature is that when a tipping point occurs, small environmental changes alter system dynamics so that previous models explaining the behaviour of the system built under one regime are no longer predictive when the regime has shifted. Although there are many reasons a model may not explain data, assuming an equilibrium state, a potential indication of a tipping point is when a model explains the data well under some conditions but then fails when environmental parameters change. Other possible signatures of tipping points include flickering between behavioural states and delayed recovery to prior states following perturbation [10].

### (b) Are critical points and tipping points equivalent?

While the terms *tipping point* and *critical point* are often used interchangeably in the literature, there are distinctions. Loosely, a critical point occurs when the stability of attractors changes. Tipping points require a quantifiable change in behavioural state as a result of minor changes in environmental parameters. This makes all tipping points critical points, but not all critical points tipping points. For example, a system moving through a critical point could have a continuous behavioural state as environmental parameters change, but a system with a tipping point would have a discontinuity in the behavioural state as a function of the environmental parameters (figure 2).

### (c) Is there hysteresis?

The existence of multiple behavioural states allows for the possibility of hysteresis—a concept often linked to tipping points in the literature [25–27]. Hysteresis is a system's lack of reversibility as environmental parameters are varied. A system exhibits hysteresis if reverting the environmental parameters in a system that has passed through a tipping point to the parameters immediately preceding the change does not cause the system to revert to the previous behavioural state. For example, once agitated, spider societies require cooling to far lower temperatures to return them to a calm state (figure 1). However, not all tipping points will exhibit hysteresis. Tipping points and hysteresis are important to consider because it changes the way that systems should be modelled. In particular, researchers may assume that their systems as reversible in parameter space but, if hysteresis is present, this is not the case.

### (d) Are there early warning signs of tipping points?

One of the most challenging aspects of tipping points is anticipating when and where they are likely to occur [22,23,28]. There are two general predictors whose presence is thought to anticipate an impending tipping point. First, increased variance in a system's internal dynamics is predicted to warn of an approaching tipping point [9,29]. Destabilized dynamics, large swings and oscillations, or flickering between states all potentially convey that the feedback that keeps a system at one attractor state is weakening, which allows the system to wander farther from the attractor. Second, the speed of recovery to baseline conditions is predicted to decrease when a system is approaching its tipping point [2,9]. This is because the strength of the feedback that maintains systems in one state decreases as a system moves toward a tipping point, and therefore the rate of recovery is slower. In behaviour, there may be other warning signs based on individual-level characteristics, or early behavioural outcomes prior to more dramatic state shifts.

## 4. Applying tipping points to animal societies

### (a) What can be learned?

Tipping points can inform our understanding of animal societies in a variety of ways. First, documenting tipping points aids our ability to forecast dramatic state shifts in animal behaviour [29]. This, in turn, can help us to predict how societies will change in response to environmental parameters, which is required for conservation [30–32]. Second, tipping points convey information about societies' comparative sensitivity to environmental parameters. The presence of abrupt tipping points, pronounced hysteresis, an inability to recover to baseline dynamics following perturbation, and large differences in behavioural states all convey that the internal dynamics driving a system are strongly nonlinear. Additionally, in the presence of tipping points, a system's responsiveness to the environment could appear deceptively small, save for the regions immediately around the tipping point. Many systems therefore may appear deceptively stable, unless one specifically interrogates the limited set of conditions that trigger the system to tip. Third, scrutinizing tipping points and their adaptive function may shed light on how social groups are capable of incredible behavioural flexibility. For example, there is evidence that societies may self-organize or evolve to keep themselves near tipping points, so that they can respond dynamically to new information or environmental challenges [27,33] and potentially maximize the adaptive advantages of both order and disorder [33]. Fourth, scrutinizing tipping points across tiers of biological organization may help us to determine whether there are generalizable features about their dynamics that bridge tiers of biological organization. Fifth, knowledge of tipping points can help guide researchers as to when a new modelling paradigm may be necessary to predict system behaviour.

### (b) How can social properties affect tipping points in social systems?

Many social properties could influence whether tipping points occur in a society. These include relatedness and group size, presence of keystone individuals, within-group behavioural diversity, group social organization, and groups' prior experience. In this section, we present a hypothetical example and then use it as a lens to pose how social properties might impact tipping points.

**Figure 3.**
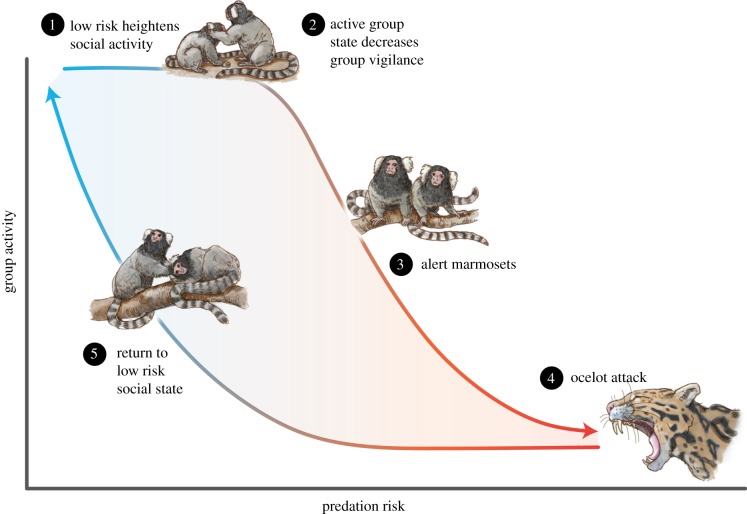
A hysteresis window depicting the relationship between group activity level (*y*-axis) in association with contrasting levels of predation risk (*x*-axis). At low levels of predation groups engage in social interactions that heighten group activity (1) but also distract groups from detecting small to moderate levels of predation risk (2). However, at some increased level of predation risk groups decrease activity and become vigilant (3), and extreme levels of risk will cause groups to go into hiding and cease activity (4). As risk dissipates, groups require a much lower level of risk to resume social activity (5).

Consider a hypothetical situation where the activity level of a group of marmosets depends on the level of predation risk (figure 3). When predation risk is low, groups are socially active and have a chance of entering distracted social states. Distracted states may emerge when one individual steals fruit or chases another individual, resulting in a competitive tit-for-tat game. Once initiated, social activity can keep a group in an active and distracted state despite mild to moderate increases in predation risk. However, at a tipping point, even a distracted group will detect heightened risk, and activity will decrease in favour of vigilance. Returning back to social activity will then require a large decrease in predation risk because vigilance renders a group sensitive to even moderate risk. Thus, under some conditions, whether a group will be active or inactive will depend on its prior state (distracted versus vigilant), creating a hysteresis window.

#### (i) Relatedness and group size

Group relatedness and size likely influence tipping points. Relatedness has an impact on a variety of social outcomes, including increased prosocial behaviour and decreased exploitation among group members [34,35]. Thus, social feedback driven by competitive interactions may be less stable between relatives [36]. Kin groups may also be more likely to share information about predation risk even at risk to themselves, for instance, via alarm calls [37,38]. Group size is also likely to impact the above scenario. Increasing group size could augment competitive interactions and keep individuals in a distracted state for longer. Larger groups may also compete more [39] and this could increase group distraction. Yet, larger groups also have more individuals with which to detect changes in the environment and share information [27,40]. The net effect of group size may therefore depend on the degree to which social interactions impede individuals' probability of detecting risk and the degree of information sharing.

#### (ii) Keystone individuals

The presence of influential individuals impacts social tipping points. For instance, the presence of leaders or reconciliatory individuals may prevent tit-for-tat feedback loops from ever starting [41,42]. In contrast, the presence of particularly aggressive, hungry, or bold individuals could increase within-group conflict [43], thus changing the environmental parameter values that result in a tipping point and the feedback strength that underlies them.

#### (iii) Behavioural diversity

More phenotypically diverse systems are predicted to be more resistant to and resilient from environmental stress [44,45]. This, in turn, will shift the timing of tipping points or cause a more linear collective response to environmental changes, i.e. eliminating tipping points altogether. The so-called *portfolio effect* predicts that more diverse groups will have increased odds that at least some constituents can endure novel environments, and therefore, maintain group-level properties [46]. In contrast, homogeneous groups run the risk of all individuals possessing the same sensitivities, making abrupt collective state shifts more likely. However, even for diverse groups, there will be some environmental parameters that cause tipping points in spite of any benefits.

#### (iv) Social organization

The social structure of our marmoset groups and the space in which the interactions occur also likely affect tipping points [29]. In groups that live in or build structures, such as nests, the geometry of these spaces can determine the kinds of interactions that individuals engage in, the degree of competition among group members, risk of predation, environmental sensitivity, and so on [47]. Nests also provide some homeostatic benefits to their residents [48], which will likely impact the susceptibility of groups to changes in environmental parameters. For groups that live in more open environments, geographical constraints such as rivers, matrix habitat, and localized resources such as fruit trees will impact individuals' position in space and therefore the structure of social networks [49]. Networks, in turn, will shape whether and how individuals interact and influence each other's behaviour [50].

#### (v) Prior experience

Whether or not social groups have previously been exposed to specific environmental parameters will likely impact their future tipping points [2,6]. For instance, prior experience with anthropogenic noise might prime a marmoset group and desensitize it to subsequent noise exposure [51]. In the related concept of cross-tolerance [52], experience with one stressor can increase the system's resistance to other stressors. The predicted outcome is similar to that of priming but differs in that stressors can appear interchangeable. A final stressor query is whether the social context of prior experience matters. For instance, the effects of prior experience may depend on whether individuals acquired their experience in isolation, in a group setting, or in a group setting that differs from their present group. The effects of such experiences will likely not be equivalent.

### (c) Organizing social tipping points

#### (i) Social scale

Social tipping points can be the *additive* outcome of tipping points occurring within each individual (*individual-level*) or the *synergistic* outcome of interactions among individuals (*group-level*). For instance, in *Polistes* paper wasps, colonies may proceed nonlinearly from a quiescent state to responsive state related to increases in disturbance. This could be an additive process, whereby the group response is the sum of each individual wasp's threshold—beyond which it moves from inaction to agitation [53]. Alternatively, a group-level response can be an emergent property, mediated by *synergistic* interactions between group members. For instance, the probability of each wasp entering an agitated state may not be independent from other wasps. The threshold to enter an agitated state may, for example, decrease when neighbours become agitated. Experiments that evaluate individual responses in isolation versus group settings, in groups of various sizes, or in groups with contrasting abilities to interact will be helpful for demonstrating the social scale at which tipping points operate.

#### (ii) Metabolic tipping points

A system may pass through a tipping point if environmental parameters drive individuals into alternative metabolic states that affect individuals' behaviour. For example, excessive heat can force social ectotherms into collective activity either to cool themselves, like collective fanning behaviour in honeybees [54], or to evacuate a nest site entirely. Another potential example of a metabolic tipping point is when excessive cold or aridity causes collective huddling to preserve heat and water in small bodied animals [55]. Metabolic tipping points can be additive or synergistic. For instance, collective huddling behaviour may enable groups of homeotherms to remain socially active in environments that exceed the thresholds of each individual in isolation [56]. In contrast, social ectotherms may exhibit a more additive response because constituents cannot share metabolic heat [57]. Other examples of metabolic tipping points can occur because of contrasting hunger levels, fat stores, hypoxia, exposure to contaminants, infection status, microbiomes, and so on.

#### (iii) Social or cognitive tipping points

Tipping points can also be mediated by social or cognitive parameters, which arise because individuals' perception of their environment has changed. For instance, cautious or flight-prone behaviour in one group member might catalyse that behaviour in another individual [58]. Alternatively, observer individuals may copy the successful foraging strategies of innovative group mates [59] or the migration routes of older individuals [42,60]. The key ingredient for these transitions is that actors are capable of observing their environment, and then adjusting their behaviour accordingly. In principle, social or cognitive state transitions can occur at different social scales as well. For example, each individual may independently learn about its environment, and therefore, the group's behaviour changes as the sum of these individual assessments. However, social interactions will often result in a synergistic shift [61].

## 5. Why study tipping points in social behaviour?

Many disciplines already use the ideas and terminology of tipping points to explore the properties of complex systems. This leads one to ask: what strengths can behavioural ecologists bring to the broader study of tipping points?

First, animal social systems provide us with the opportunity to observe interactions between individual-level and group-level tipping points. Although this review pertains to social tipping points in the dynamics of whole societies, individual organisms are themselves complex living systems with metabolic processes that can undergo tipping points in response to environmental parameters [62,63]. One can therefore probe the scale at which tipping points occur by evaluating behavioural dynamics in response to environmental drivers when individuals are in isolation versus group settings or across groups of various size. Linking tiers of multilevel tipping points is a problem already faced by the tipping point literature on communities and ecosystems [6,63,64], but one fears that the problem of scale (individual versus population versus community versus ecosystem) might be intractably great in such systems. Social tipping points in animal societies might therefore serve as a convenient intermediate ground in which to develop and critically evaluate theory on multilevel tipping points. Such individual versus group-level comparisons do not have clear analogues in the application of tipping points in the physical sciences. This opens the door to new lines of empirical inquiry and theory.

Second, behavioural ecologists have the ability to create large numbers of experimental systems [65,66] and manipulate environmental parameters thought to cause tipping points [13], thus allowing cause-effect inferences that elude purely theoretical studies or correlative studies on other living systems. General ecologists have used the tipping point framework to explore contrasting ecosystem dynamics [2], shifts in community composition and functioning [4,8], and decreases in the viability of imperilled wildlife populations [31,32]. Engineering such systems or altering them experimentally with a high degree of replication is often impossible or unethical. For many social systems, this is not so.

Third, using the tipping point framework has the potential to foster crosstalk between the kinds of questions asked by behavioural ecologists and investigators interested in other kinds of complex systems. The notion that similar principles might underlie the presence, severity, timing, and recoverability of tipping points across contrasting physical and living systems is intriguing, and behavioural ecologists are poised to enter this dialogue with precision.

Finally, animal societies raise our consciousness to the presence of asymmetrical interaction rules, and therefore promise to inspire new kinds of tipping point models, which often assume that interaction rules are simple, symmetrical, and invariant. In animals, we know that individuals differ from each other in their attributes, the ways in which they interact, and the social influence they exert over their groups. While behavioural ecologists have potentially much to gain from the tipping point literature, the intellectual exchange promises to be bidirectional.

## 6. Conclusion

Many living systems exhibit drastic state shifts in response to small changes in environmental parameters. We argue here that animal societies—like other kinds of living systems—can be subject to tipping points and that a better understanding of tipping point dynamics can help us to predict changes in sociality and behaviour. Behavioural ecologists interested in such dynamics are poised to contribute novel insights, both theoretically and empirically. The insights gleaned from such studies have the potential to generate crosstalk between fields of ecology that typically operate independently. The tipping point framework in turn offers us (behavioural ecologists) a variety of opportunities. First, the tipping point framework asks us to re-examine familiar topics—information spread, collective action, group formation/disbandment, etc.—from a new perspective, which opens up new flavours of inquiry. Second, tipping points draw our attention to possible connections between the dynamics of social systems and other kinds of complex living systems—highlighting the opportunity for generalizing principles across tiers of biological organization. Third, the tipping points framework draws our attention to ideas from other subdisciplines of ecology and allows us to critically evaluate these ideas in a new context. Finally, understanding tipping points is of conservation importance for multiple tiers of biological organization, which permits basic researchers to probe tipping points while simultaneously collecting data that could prove useful for applied scientists. This incipient field is therefore ripe for creation and entry, and there is much for us to discover together.

## Data accessibility

This article has no additional data.
